# Suicide prevention during disasters and public health emergencies: a systematic review

**DOI:** 10.3389/fpubh.2024.1338099

**Published:** 2024-02-06

**Authors:** Lennart Reifels, Karolina Krysinska, Karl Andriessen

**Affiliations:** Centre for Mental Health, Melbourne School of Population and Global Health, The University of Melbourne, Parkville, VIC, Australia

**Keywords:** suicide, suicide prevention, disasters, public health emergencies, pandemics

## Abstract

**Background:**

Disasters and public health emergencies increasingly affect populations around the world, posing significant wide-ranging challenges for societies as well as for effective public health and suicide prevention. Intervention research is essential to inform evidence-based responses. Yet, despite evident public concern and growing research interest in heightened suicide risks and impacts, little is known about effective suicide prevention interventions in these contexts. We conducted a systematic review to examine the outcomes of suicide prevention strategies implemented in disasters and public health emergencies.

**Methods:**

We searched five databases (Medline, Embase, PsycINFO, Web of Science, PTSDpubs) from inception to December 2022 for peer-reviewed quantitative studies that reported relevant intervention outcomes (changes in the frequency of suicide, suicide attempts, self-harm) for populations affected by disasters and public health emergencies. We assessed the quality of eligible studies using the Quality Assessment Tool for Quantitative Studies, and distilled review findings through narrative synthesis. The study protocol was registered with PROSPERO (CRD42021276195).

**Results:**

Ten eligible and mostly observational studies were included in this review, which examined a range of universal, selective, and indicated interventions. Three of five studies of interventions in public health emergencies indicated the potential effectiveness and buffering effects of generic disaster related mental health support, access to urban parks, as well as the beneficial role of video-enabled tablets in facilitating treatment access and outcomes. Similarly, three of five studies of interventions in disaster contexts provided evidence of the beneficial role of universal economic security measures, national gun laws and buy back schemes, and volunteer-delivered mental health support. Overall, four of six studies with favorable outcomes examined interventions specifically deployed in disaster or public health emergency contexts, whereas two studies examined ongoing existing interventions. Three studies, respectively, of suicide prevention focused interventions or generic interventions reported favorable outcomes. The quality of included studies was variable, with two studies being rated as ‘strong’, four studies rated as ‘moderate’, and four studies rated as ‘weak’.

**Conclusion:**

Notwithstanding the limited scope and variable quality of published evidence, our review findings highlight the breadth of interventions that have been applied in such contexts with some success. There is a need for further research on effective interventions and intervention adaptations to inform evidence-based suicide prevention responses to disasters and public health emergencies.

**Systematic review registration:**

https://www.crd.york.ac.uk/prospero/display_record.php?ID=CRD42021276195, PROSPERO ID CRD42021276195.

## 1 Introduction

Suicide is a major global public health concern that calls for effective and concerted preventive intervention (1). Each year, approximately 700,000 people die by suicide (2). Suicide is a complex phenomenon which is influenced by a range of contextual factors that include prevailing social, socio-economic, and environmental conditions. These can no longer be relied upon as immutable or enduring, and are rather in themselves often subject to ever more rapid change and disruption (3, 4).

Disasters and public health emergencies (such as pandemics and epidemics) are increasingly affecting populations around the world (5, 6), with significant wide-ranging implications for societies, human livelihoods, health, and wellbeing, as well as public health (7). Many established proximal and contextual risk factors for suicide (8, 9), such as adverse life events, losses, financial stressors, social isolation, reduced social support and healthcare access, are present or elevated in the wake of disasters (10) and public health emergencies (11). While suicidal behavior trajectories can vary following disasters (with some indications of an early drop and delayed increase pattern) (12), overall suicide rates have been found to increase among whole populations and male subpopulations (13).

Suicidality has also been of significant public concern during protracted public health emergencies, such as prominently in the unfolding Covid-19 pandemic (14). National suicide rates did not increase in the first 15 months of the COVID-19 pandemic (15, 16). Yet, systematic reviews and expert guidance highlight a continued need for vigilance (17) in view of elevated distress levels among affected populations (18), rising self-harm presentations among young people (19), heightened suicidality risk among COVID-19 patients (18), increased demand for non-acute support services (20, 21), and strain on frontline healthcare workers (22). All of these have implications for targeted suicide prevention efforts during these challenging and disruptive circumstances (23).

Although the broader evidence base for effective suicide prevention approaches across the spectrum of universal, selective, and indicated interventions is consolidating (24, 25), little is known about the outcomes of suicide prevention activities during disasters and public health emergencies. In fact, much research to date has focused on the epidemiology of suicidality in such contexts, while there is an urgent need for research on interventions (18, 26) to inform evidence-based suicide prevention responses (27). What types of routine existing or disaster-specific suicide prevention interventions have been found to be effective in such contexts therefore remains an open question. To our knowledge, this is the first systematic review to examine the outcomes of suicide prevention strategies implemented in the context of disasters and public health emergencies.

## 2 Methods

This systematic review is presented following the Preferred Reporting Items for Systematic reviews and Meta-Analyses (PRISMA) (28) and the review protocol was prospectively registered with PROSPERO (CRD42021276195).

### 2.1 Eligibility criteria

Studies meeting the following eligibility criteria were included:

- Population: Populations affected by disasters (marked by natural, human-induced, technological hazards) or public health emergencies (including epidemics, pandemics, infectious disease outbreaks)
- Intervention: Any type of strategy, program, intervention with an explicit focus on suicide prevention or postvention (or other intervention reporting suicidality/self-harm outcomes) for populations affected by disasters or public health emergencies
- Context: New, existing, or adapted suicide prevention strategies, programs, interventions implemented in the context of disasters and public health emergencies (including rapid or slow onset events, and protracted emergencies)
- Outcomes: Changes in the frequency of suicide attempts, suicide deaths, or self-harm (reported by any measure)
- Study design: Quantitative studies (or quantitative components of mixed-method studies)
- Comparator: Intervention studies including any comparator (e.g., before/after, by sub-group, by intervention type)
- Article type: English language, peer-reviewed, empirical studies, human

Exclusion criteria:

- Context: Euthanasia, assisted dying, warfare, armed conflict, civil unrest, economic crisis
- Outcomes: Non-suicidal self-injury, suicidal ideation, composite suicidality measures
- Study design: Qualitative studies
- Article type: Commentaries, editorials, conference abstracts, dissertations/theses, grey literature

### 2.2 Information sources

We searched five literature databases, Medline (Ovid), Embase (Ovid), PsycINFO (Ovid), Web of Science (Clarivate), and PTSDpubs/PILOTS (ProQuest), and conducted additional reference list screening of selected review papers and forward citation searches of relevant study protocols.

### 2.3 Search strategy

All databases were initially searched on 14 January 2022 (from database inception to search date), and the entire search was updated on 7 December 2022. The searches used a combination of MeSH terms and database specific key words regarding the three domains of outcome (suicidality and self-harm), context (disaster, public health emergency, infectious disease outbreak), and intervention. Full search strategies for all databases and definitions of key terms are included in the Supplementary Material.

### 2.4 Selection process

A two-stage record screening and study selection process was undertaken by two researchers, using EndNote. First, two researchers (LR, KK) independently screened the titles and abstracts of records to identify potentially eligible studies. Second, two researchers (LR, KK) independently assessed the full texts of potentially eligible studies against the review inclusion and exclusion criteria to identify studies to be included in the review. Any disagreements were resolved through discussion or referral to a third researcher (KA).

### 2.5 Data extraction and synthesis

One researcher (LR) extracted the following data from included studies using a piloted data extraction tool (set up in MS Excel), and all data were checked by a second researcher (KK):

- Study characteristics (title, primary author, journal, publication year, study aim, design, language)
- Sample characteristics (sample size, mean age, age range, gender, ethnicity, country, study period)
- Type of disaster exposure (disaster type, year, exposure measure)
- Intervention characteristics (intervention type, modality, setting, timing relative to disaster, new/existing/adapted)
- Outcome measures (for suicide, suicide attempts, self-harm)
- Results (main findings, effect sizes, limitations)

Study findings were distilled through narrative synthesis (including tabulation and grouping by context and intervention subgroups). Substantial study heterogeneity (regarding interventions, outcome measures, target populations, and settings) precluded formal meta-analysis and calculation of pooled effect estimates.

### 2.6 Study quality appraisal

We used the Quality Assessment Tool for Quantitative Studies to assess the methodological quality of the included studies (29). The instrument includes six components (selection bias, study design, confounders, blinding, data collection methods, and withdrawals and dropouts) to be scored as strong, moderate, or weak. A study was rated ‘strong’ if none of its components was rated ‘weak’. A study was rated ‘moderate’ if one of the components was rated ‘weak’, and it was rated ‘weak’ if two or more of its components were rated as ‘weak’ (29). The instrument also assesses the integrity of the intervention and analyses (e.g., appropriate statistical methods). Two researchers (KK, KA) independently assessed the quality of the included studies. There was substantial agreement between the two researchers (*κ* = 0.64), and they resolved any disagreement by discussion, or by referral to a third researcher (LR).

## 3 Results

### 3.1 Study selection

**Figure 1:**
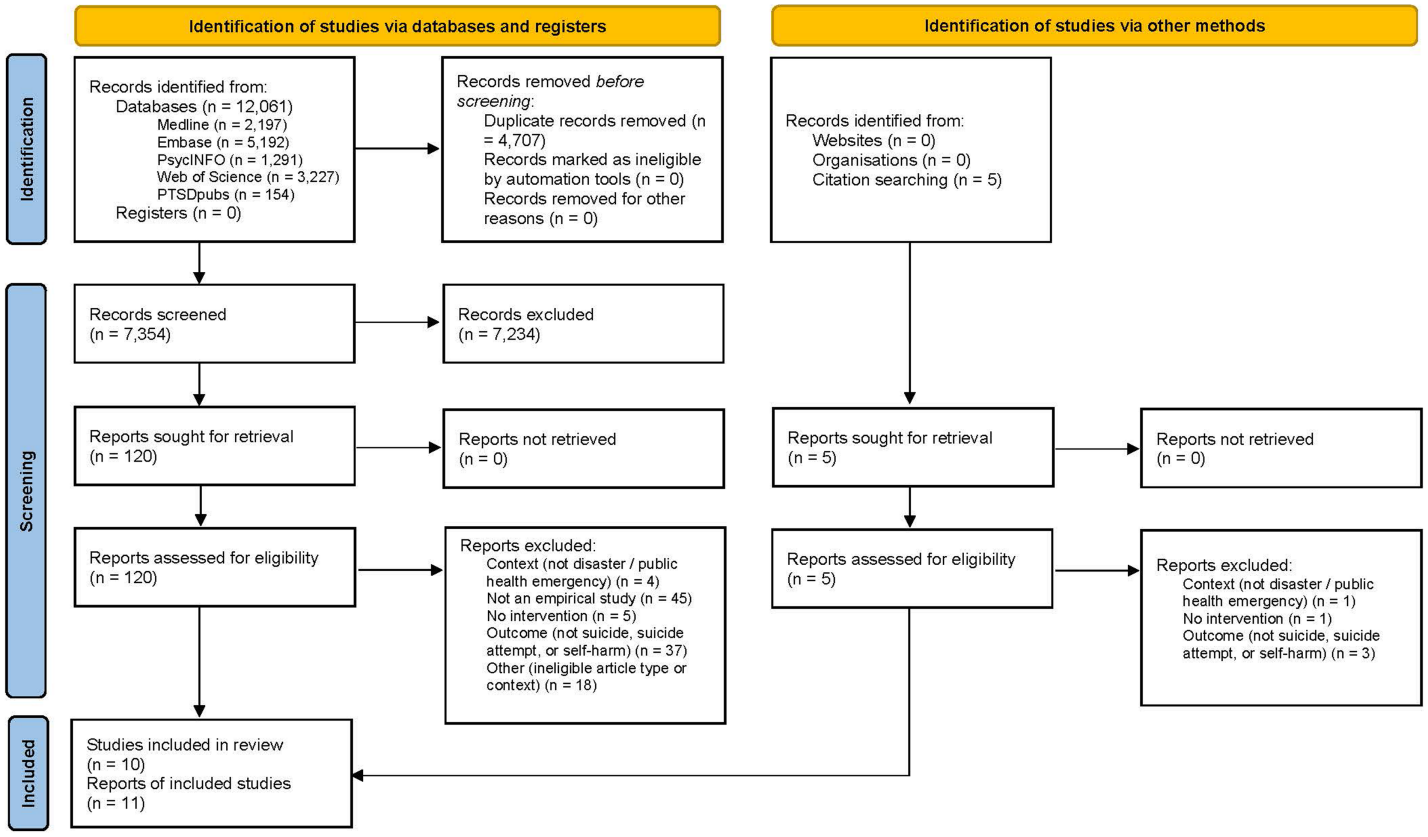
PRISMA flow chart for study selection.

The study selection process is presented in the flow chart diagram (Figure 1). Database searches yielded a combined total of 12,061 records. Following removal of 4,707 duplicates, 7,354 records were initially screened by title and abstract, leading to the exclusion of 7,234 records. Full-text eligibility assessment of 120 remaining reports resulted in the exclusion of 109 reports (including 45 reports not constituting empirical studies, 37 reports not examining relevant outcomes, 18 reports reflecting ineligible article types, 5 reports not examining an intervention, and 4 reports lacking a relevant context). Additional reference list and forward citation searches identified 5 potentially eligible reports, which were excluded at full-text assessment at these did not meet the inclusion criteria. Ultimately, 11 reports relating to 10 studies were included in the systematic review (30–40). Of the two reports referring to the same study (38, 39), only the most recent was considered (38), as the other did not provide additional relevant data.

### 3.2 Study characteristics

Table 1 presents the characteristics of included studies, published between the years 2008 and 2022. In terms of geographical location, five of the ten studies were conducted in Asia (India, Japan, South Korea), two studies, respectively, in North America (Canada, U.S.A.) and Oceania (Australia), and one study in Europe (U.K.). Five studies examined interventions in the context of public health emergencies (Covid-19 pandemic, 2009 H1N1 swine flu pandemic, 2015 Middle East Respiratory Syndrome or MERS epidemic), while the other five studies focused on disaster contexts (marked by mass shootings, tsunamis, or multiple hazards). Most study designs were observational in nature, including cohort studies, time series, or interrupted time series analyses, and only one study adopted a randomized controlled trial design.

**Table 1:** Study characteristics and findings.

Author	Country	Context (PHE / disaster)	Study design type	Sample / participants	Intervention	Comparator	Outcome / effect measure	Main findings
Challinor et al. (40)	United Kingdom	PHE (COVID-19 pandemic)	Cohort	Patients at high-secure psychiatric hospital wards (*n* = 118) Gender (female): 0% Study period: 2020–2021	Psychiatric hospital treatment	Ward type (Mental illness vs. personality disorder, and high vs. medium dependency)	Self-harm Monthly incidence (change)	The higher self-harm incidence during the initial pandemic stage (April–June 2020), particularly on high-dependency PD wards (coinciding with 1^st^ lockdown and ward containment measures), subsided thereafter (coinciding with safe resumption of TAU on wards from June 2020).
Gujral et al. (31)	United States	PHE (COVID-19 pandemic)	Cohort	Rural veterans with a history of mental health care use (n = 471,791), including 13,180 tablet recipients Age (mean): 61.2 years Gender (female): 12% Ethnicity: 2% Hispanic, 97% not Hispanic, 1% unknown Study period: 2019–2022	Video-enabled tablets	Control group (not receiving tablets)	VA suicide behavior and overdose reports (SBORs) Difference-in-difference coefficients	Tablets were associated with a 22% decrease in SBORs (monthly coefficient − 0.0011; 95% CI −0.0016 to −0.0005), and 168 fewer suicide behavior reports per year. For the subcohort of rural veterans at high risk of suicide, tablets were associated with a 22% decrease in SBORs (monthly coefficient − 0.0075; 95% CI −0.125 to −0.0026), and 96 fewer suicide behavior reports per year.
Kim et al. (30)	South Korea	PHE (2009 H1N1 pandemic; 2015 MERS epidemic)	Cohort	Study area population (n = 386,125) Study period: 2003–2018	Urban parks	*Per capita* park area by city-county area	Suicide Rates	Urban parks functioned as a mitigator to prevent increasing suicide rates in the pandemic (especially if associated with economic shocks). With every 1m^2^ per person increase in park area, the suicide rate per 100,000 people decreased by 0.38.
Klim-Conforti et al. (37)	Canada	PHE (Covid-19 pandemic)	RCT	Grade 7–8 students in urban schools (n = 430; 200 intervention, 230 control) Age (range): 11–14 years Gender (female): 61.6% Study period: 2019–2020	Harry Potter-based CBT skills training	Waitlist controls (regular curriculum)	Suicide attempts Pre/post mean difference	There was no significant difference in respective changes in suicide attempts between intervention (Mean 0.06, SD 0.20) and control groups (Mean 0.04, SD 0.18), (*t* − 0.90, df 406, *p* = 0.37).
Orui et al. (33)	Japan	PHE (COVID-19 pandemic) Disaster (earthquake, tsunami, nuclear accident)	Cohort	Suicides in affected areas (n = 716), unaffected areas (n = 1,678), and nationally (n = 304,162) during study period Study period: 2009–2020	Disaster mental health interventions	Affected vs. non-affected areas vs. national	Suicide Standard Mortality Ratio (SMR)	The SMR rose to 1.20 (95% CI 1.02–1.47) in unaffected areas in 2020 (during the pandemic) compared to 0.98 (95% CI 0.74–1.29) in intervention areas.
Chapman et al. (38) Chapman et al. (39)	Australia	Disaster (mass shooting)	ITS	National firearm (n = 12,247) and non-firearm (n = 64,623) suicides during study period Study period: 1979–2013	Gun laws and buyback scheme	Pre/post	Firearm suicide Annual rate decline (trend ratio)	The annual rate decline accelerated from 3 to 4.8% following intervention (trend RR 0.981; 95% CI 0.970–0.993); indicating a step change in the level of firearm suicides (RL = 0.652; 95% CI 0.582–0.731); with no indication of substitution to other lethal methods.
Lee et al. (36)	Australia	Disaster (mass shooting)	TSA	National firearm / non-firearm suicides during study period Study period: 1915–2004	Gun laws and buyback scheme	Pre/post	Firearm suicide Structural breaks in growth rates	There was no evidence of a structural break in firearm suicide growth rates around the time of the intervention; and no indication of substitution effects.
Matsubayashi et al. (35)	Japan	Disaster (earthquake, tsunami, nuclear accident)	Cohort	Suicides in study regions during study period Age: ≥ 20 years Study period: 2002–2019	Economic security measures	Severely damaged vs. unaffected prefectures	Suicide Rate change	A 1% increase in per-capita local government expenditure was associated with a 0.104% decrease in the suicide rate among men aged 20–39 years and a 0.073% decrease in men aged 40–64 years.
Nakanishi et al. (34)	Japan	Disaster (earthquake, tsunami, nuclear accident)	ITS	National suicides during study period (*n* = 597,007) Age (mean): 52.9 years Gender (female): 29.2% Study period: 1996–2016	Suicide prevention act	Pre/post	Suicide Rate change (trend difference)	Overall suicide trends were not interrupted by the Act: change 0.055 [−0.037, 0.147], trend −0.001 [−0.003, 0.001], trend difference − 0.0004 [−0.003, 0.002].
Vijayakumar et al. (32)	India	Disaster (tsunami)	Cohort analytical	Bereaved tsunami survivors (*n* = 102; 45 intervention, 57 control) Age (mean): 38.2 years Gender (female): 51% Study period: 2004–2006	Trained volunteer delivered mental health support	Bereaved participants from control site (not receiving intervention)	Suicide attempts Pre/post change in counts	Significantly less suicide attempts were observed in the intervention group (FET *p* = 0.02), reducing from 6 to 0, compared to the control group (7 to 3).

Chapman et al. (38, 39) were regarded as two reports from the same study and only the most recent report was considered. PHE, public health emergency; ITS, interrupted time series; TSA, time series analysis; FET, fisher’s exact test; VA, Veteran’s affairs; TAU - treatment as usual.

The examined intervention types included psychiatric hospital treatment, disaster related mental health support, video-enabled tablets, CBT-skills training, and varied universal measures (including national gun laws and buy back scheme, a suicide prevention act, economic security measures, as well as urban parks). Interventions were either ongoing existing measures (30, 31, 34, 37, 40) or specifically deployed in disaster contexts (32, 33, 35, 36, 38). Some interventions included an explicit focus on suicide prevention (31, 33, 34, 37), whereas other generic interventions did not (30, 32, 35, 36, 38, 40). Target groups for interventions were whole populations (30, 34, 36, 38), people in disaster affected areas (33, 35), rural veterans (31), psychiatric hospital patients (40), school students (37), and bereaved tsunami survivors (32).

Studies reported intervention outcomes in terms of suicide (30, 33–35), firearm suicide (36, 38), suicide attempts (31, 32, 37), and self-harm (40). Study comparators included pre/post comparisons (34, 36, 38), different hospital ward types (40), affected versus non-affected areas (33, 35), control groups not receiving the intervention (31, 32, 37), and variations in per capita park area (30).

### 3.3 Intervention outcomes

#### 3.3.1 Outcomes by context

Of the five studies examining interventions in public health emergency contexts, three observational studies reported a reduction in suicides and suicide attempts (30, 31, 33), one observational study provided mixed results (40), and one RCT found the intervention not effective in reducing suicide attempts (37). Specifically, the study by Orui et al. (33) examined suicide rates during the Covid-19 pandemic in areas that had previously been affected by the Japanese triple (earthquake, tsunami and nuclear) disaster, and which continued to receive ongoing disaster mental health support, by comparison to unaffected areas not receiving such support. This study found that the suicide related standard mortality ratio rose to 1.20 in unaffected areas during the pandemic and remained relatively stable at 0.98 in intervention areas, indicative of a possible buffering effect of existing support. The study by Kim et al. (30) reported that urban parks functioned as a mitigator of increasing suicide rates in the 2009 H1N1 pandemic in that with every 1m^2^ per person increase in park area, the suicide rate per 100,000 people decreased by 0.38. The study by Gujral et al. (31) found that escalated distribution of video-enabled tablets among rural US veterans during the Covid-19 pandemic was associated with increased mental health service use and a 22% decrease in the likelihood of suicide behavior. Challinor et al. (40) monitored self-harm incidents among patients of different secure psychiatric hospital wards during the Covid-19 pandemic. Study findings indicated that a higher self-harm incidence during the initial pandemic stage particularly on high-dependency personality disorder wards (which coincided with the first lockdown and ward containment measures), subsided thereafter (coinciding with the safe resumption of treatment as usual). Finally, the RCT conducted by Klim-Conforti et al. (37) during the Covid-19 pandemic indicated no benefits of CBT-skills training in reducing suicide attempts among urban school students when compared to a control group not receiving the intervention.

Three of five studies examining interventions in disaster contexts provided encouraging results (32, 35, 38) while two studies provided no evidence of intervention effectiveness (34, 36). The interrupted time series study by Chapman et al. (38) indicated a step change and accelerated decline in annual firearm suicides with the introduction of the national gun laws and buyback scheme in the wake of Australia’s worst mass shooting. By contrast, the time series analysis by Lee et al. (36) conducted in the same disaster context provided no evidence of a structural break in firearm suicide growth rates around the time of the intervention. The cohort study by Matsubayashi et al. (35) examined economic security measures and suicide rates in the context of the Japanese triple disaster, finding that a per-capita increase in local government expenditure was associated with a decrease in the suicide rate among men. The interrupted time series study by Nakanishi et al. (34) conducted in the same context provided no evidence that suicide trends were interrupted by the introduction of a national suicide prevention Act. Finally, the cohort-analytical study by Vijayakumar et al. (32) examined volunteer delivered mental health support among bereaved tsunami survivors, reporting significantly less suicide attempts in the intervention group compared to a control group.

#### 3.3.2 Outcomes by intervention

Only two of five studies examining ongoing existing interventions (i.e., urban parks and video-enabled tablets) (30, 31) and four of five studies of interventions specifically deployed in disaster or public health emergency contexts (i.e., gun laws and buyback scheme, economic security measures, disaster mental health interventions, volunteer delivered mental health support) (32, 33, 35, 38) reported findings indicative of intervention effectiveness. Similarly, three of five studies, respectively, examining suicide prevention focused interventions (31–33) or generic interventions (30, 35, 38) reported reductions in suicidality outcomes. Two studies which examined the same universal intervention (gun laws and buy back scheme), using varying time series designs, provided contradictory results in terms of intervention effectiveness (36, 38).

### 3.4 Study quality

The study quality assessment is outlined in the Supplementary Material. The overall quality of included studies was variable, with two studies being rated as ‘strong’ (34, 38), four studies rated as ‘moderate’ (30, 33, 35, 36), and four studies rated as ‘weak’ (31, 32, 37, 40). The quality domains rated most strongly across studies were selection bias and data collection methods. Four studies with overall ‘weak’ quality ratings were each rated ‘weak’ on two quality domains in terms of blinding (31, 32, 37), confounders (32, 40), selection bias (37), data collection methods (40), or withdrawals and dropouts (31).

## 4 Discussion

This systematic review identified 10 studies which reported changes in suicidality outcomes associated with interventions conducted in the context of disasters and public health emergencies. Taken together, the limited scope of published evidence, variable study quality, and diversity of interventions and contexts precluded firm assessments of intervention effectiveness. Yet, our review findings provide several valuable insights that can help to inform future suicide prevention practice and research in these increasingly pervasive and challenging contexts (41).

Overall, our findings highlight the breadth of interventions that have been applied and studied in these contexts (including explicit suicide prevention and generic interventions, ongoing existing and specifically deployed interventions, across the full spectrum of universal, selective, and indicated intervention). Notwithstanding evidence limitations, the included studies provided some indication of favorable intervention outcomes in the context of pandemics and disasters.

Two cohort studies of moderate quality provided preliminary evidence of potentially mitigating effects of urban parks (30) and ongoing disaster mental health interventions (33) on suicide rates during pandemics. Yet, neither study design permitted firm causal attribution or fully accounted for relevant confounders. A third cohort study indicated that escalated distribution of video-enabled tablets among veterans during a pandemic improved mental health service engagement and reduced suicidal behavior (31). Whilst rating positively on several quality domains, this study did not account for blinding and dropouts. By contrast to the broader evidence on effective school-based suicide prevention (42, 43), the study adopting the most robust RCT design (but of weak overall quality due to potential selection bias and blinding concerns) found school-based CBT-skills training not to be effective in reducing student suicide attempts during a pandemic (37). Findings of a fourth cohort study remained inconclusive but indicated fluctuations in the self-harm incidence among secure psychiatric hospital patients in alignment with adapted service delivery during pandemic lock down restrictions (40).

Within disaster contexts, two cohort studies (32, 35) highlighted that increased economic security measures in terms of local government spending were associated with decreased suicide rates among men, while volunteer delivered mental health support was associated with decreased suicide attempts among bereaved survivors. Two studies examining the same national gun laws and buy back scheme provided contradictory results, with one study of strong quality (and backed by two reports) indicating positive effects (38), whereas the other study of moderate quality and employing a differing analysis did not (36). Reduced suicide rates previously associated with the introduction of national suicide prevention programs and acts (44, 45), were not observed during disasters (34).

It is noteworthy that studied interventions included hardly any designated suicide prevention interventions specifically designed for disasters or public health emergencies (33). Nevertheless, findings provided some indication of potential suicide prevention co-benefits of generic universal interventions, such as gun laws, economic security measures, and green spaces. While broader disaster mental health intervention frameworks and guidelines exist (46), these currently provide little guidance on suicide prevention. In the absence of an evidence base for designated interventions in such contexts, it therefore remains reasonable to assume that generic evidence-based suicide prevention interventions that have been effective under other circumstances (24, 25) should also have the best chance of unfolding those impacts during disasters and public health emergencies. Yet, the reasons for why their impacts and effectiveness may be hampered in these contexts are manifold, including the destructive and disruptive nature of disasters and pandemics that can simultaneously affect and overwhelm many realms of society, and which may necessitate nimble adaptations in suicide prevention programs or services (23). In fact, four interventions showed some evidence of planned reactive adaptations in these contexts (31, 33, 35, 40) that were either aimed at temporarily decreasing the scale and changing the mode of delivery to reduce virus transmission and ensure health and safety during pandemics (33, 40), or at increasing the overall scale, access and reach of interventions during pandemics and disasters (31, 35). Beyond adaptations to ensure the continuity, access and safety of existing interventions, the timing of designated interventions also deserves consideration within a broader public health approach, as systematic reviews indicate the need for a long-term perspective in view of commonly delayed suicidality increases (12) and protracted secondary stressors in such contexts (47). The integration of designated provisions to recognize and address heightened suicide risks in disaster mental health frameworks (46, 48) and pandemic response plans (49) is key to advancing future preparedness and responses.

Several intervention studies with favorable outcomes addressed known risk and protective factors for suicide of relevance to disasters (10) and public health emergencies (11) through mechanisms aimed at enhancing economic security, health care access, means restriction, psychosocial support, and green space access of affected populations. Research on the effectiveness of ongoing existing or adapted interventions as well as the development of event-specific interventions specifically targeting relevant risk and protective factors of suicide in these contexts therefore provide promising avenues to advance the field.

Research is essential to strengthen the evidence base on effective interventions (26) and inform evidence-based responses (27), but can also be challenging to conduct (50, 51) and itself be disrupted by disasters and pandemics, as was evident in one instance (37). While conducting robust gold standard RCTs may not always be feasible in these circumstances, well-controlled cohort studies and time series analyses provide feasible research methods that afford a level of rigor. When researching the impacts of ongoing interventions, findings can also be harder to interpret, as the onset of a disaster essentially constitutes a new secondary exposure or intervention, which complicates the interpretation of primary intervention effects. It is therefore essential that studies clearly capture the nature and level of disaster exposure among affected target populations and settings. Research on adapted interventions will equally benefit from clearly documenting intervention adaptations (52, 53) and from assessing adaptation outcomes (54) along with overall effectiveness outcomes (55).

### 4.1 Study limitations

Study findings should be considered in light of certain limitations regarding the available evidence and review process. These include the limited scope of published evidence and variable quality of studies. Importantly, many observational study designs did not permit firm casual attribution of intervention effects. Considerable study heterogeneity (regarding interventions, target groups, and contexts) also precluded formal quantitative synthesis and meta-analysis.

The systematic review process was based on a comprehensive literature search and rigorous study selection strategy but limited to peer-reviewed literature and quantitative empirical studies published in English. It did not consider grey literature, qualitative studies, or non-English language publications. It is further possible that the search strategy may have missed some relevant studies (e.g., of routine interventions continuing throughout disasters and pandemics) if these did not make explicit reference to such contexts. While publication bias was not formally assessed, it is conceivable that intervention studies that were either interrupted by such events or which produced negative or less favorable results in such contexts, were less likely to be published, and were therefore not available for this review.

## 5 Conclusion

Notwithstanding the limited scope and variable quality of published evidence, our review findings highlight the breadth of interventions that have been applied in such contexts with some success. There is a need for further research on effective interventions and intervention adaptations to inform evidence-based suicide prevention responses to disasters and public health emergencies.

## Data availability statement

The original contributions presented in the study are included in the article/Supplementary material, further inquiries can be directed to the corresponding author.

## Author contributions

LR: Conceptualization, Data curation, Formal analysis, Funding acquisition, Methodology, Validation, Visualization, Writing – original draft, Writing – review & editing. KK: Conceptualization, Formal analysis, Methodology, Validation, Writing – review & editing. KA: Conceptualization, Formal analysis, Methodology, Validation, Writing – review & editing.
