# Supplementary material for: Suicide prevention during disasters and public health emergencies: a systematic review
Source: Front Public Health. 2024 Feb 6;12:1338099. doi: 10.3389/fpubh.2024.1338099 (PMC10876787; doi:10.3389/fpubh.2024.1338099)
Supplement: Supplementary file 1 [file Table_1.DOCX]

Supplementary Material

**Table S1 Full search strategy Medline (Ovid).**

Databases: Ovid MEDLINE(R) and Epub Ahead of Print, In-Process, In-Data-Review & Other Non-Indexed Citations and Daily

| **#** | **Searches** |
| --- | --- |
| 1 | (suicid* or self-harm or "self harm" or self-injur* or "self injur*" or parasuicid* or auto-mutilation or automutilat* or "auto mutilat*" or "self mutilat*" or self-immolat* or "self immolat*" or self-poisoning or "self poisoning" or "intentional overdose" or "intentional drug overdose" or self-cutting or "self cutting").mp. [mp=title, abstract, original title, name of substance word, subject heading word, floating sub-heading word, keyword heading word, organism supplementary concept word, protocol supplementary concept word, rare disease supplementary concept word, unique identifier, synonyms] |
| 2 | exp suicide/ or suicide.mp. |
| 3 | exp Self-Injurious Behavior/ or self-harm.mp. |
| 4 | 1 or 2 or 3 |
| 5 | (disaster* or catastrophe* or mass-casualty or "mass casualty" or "natural hazard*" or "extreme event*" or "extreme weather event*" or "severe weather" or "heat wave*" or heatwave or drought* or "cold wave*" or cyclone* or hurricane* or tornado* or storm* or cloudburst or flood* or tsunami* or bushfire* or wildfire* or "wild fire" or brushfire or "brush fire" or earthquake* or volcan* or eruption or lahar or "lava flow" or "ash fall" or avalanche* or landslide* or mudslide* or bioevent or bio-event or bio-disaster or "humanitarian emergenc*" or stampede* or terrorism or "terrorist attack*" or "mass violence" or "mass shooting" or "school shooting" or "mass killing*" or explosion* or bombing* or fire or "nuclear accident*" or "nuclear incident*" or "nuclear power plant" or "radiation accident*" or "radiation incident" or CBRNE or CBRN or "oil spill" or "chemical spill" or "structural collapse" or "mine collapse" or "building collapse" or natech or "transport accident*" or "transportation accident*" or "aviation accident*" or "plane crash" or "rail accident*" or derailment or "train crash" or shipwreck or capsize* or "marine incident*" or "financial shock" or "financial crisis" or recession* or "economic downturn*" or "economic depression" or "economic crisis" or austerity).mp. [mp=title, abstract, original title, name of substance word, subject heading word, floating sub-heading word, keyword heading word, organism supplementary concept word, protocol supplementary concept word, rare disease supplementary concept word, unique identifier, synonyms] |
| 6 | exp disasters/ |
| 7 | Explosions/ or Fires/ or Structure Collapse/ or Tsunamis/ or Volcanic eruptions/ or Extreme Heat/ or Extreme Weather/ or Biohazard Release/ or Chemical Hazard Release/ or exp Radioactive Hazard Release/ or Accidents, Aviation/ or Accidents, Traffic/ or exp terrorism/ or Bombs/ or Economic Recession/ |
| 8 | 5 or 6 or 7 |
| 9 | ("public health emergenc*" or pandemic* or epidemic* or outbreak*).mp. [mp=title, abstract, original title, name of substance word, subject heading word, floating sub-heading word, keyword heading word, organism supplementary concept word, protocol supplementary concept word, rare disease supplementary concept word, unique identifier, synonyms] |
| 10 | Emergencies/ or exp disease outbreaks/ |
| 11 | 9 or 10 |
| 12 | (covid-19 or coronavirus or 2019-ncov or sars-cov-2 or covid* or ncov or hcov or betacoronavirus or Sars-Cov-3 or influenza or H1N1 or H2N2 or H3N2 or H7N9 or "asian flu" or "asian influenza" or "hong kong flu" or "hong kong influenza" or "spanish flu" or "spanish influenza" or ebola or SARS or "severe acute respiratory syndrome" or MERS or Mers-cov or "middle east respiratory syndrome" or "Zika virus" or arbovirus* or dengue or "swine flu" or "swine influenza" or "yellow fever" or "hemorrhagic fever" or "haemorrhagic fever" or encephalitis or plague or smallpox or chikungunya or cholera or "crimean-congo haemorrhagic fever" or "crimean congo haemorrhagic fever" or "crimean-congo hemorrhagic fever" or "crimean congo haemorrhagic fever" or "hendra virus" or "lassa fever" or marburgvirus or marburg virus disease or marburg or meningitis or monkeypox or "nipah virus" or "rift valley fever" or tularemia or tularaemia).mp. [mp=title, abstract, original title, name of substance word, subject heading word, floating sub-heading word, keyword heading word, organism supplementary concept word, protocol supplementary concept word, rare disease supplementary concept word, unique identifier, synonyms] |
| 13 | COVID-19/ or Sars-Covid-2/ |
| 14 | 12 or 13 |
| 15 | 11 and 14 |
| 16 | 4 and 8 |
| 17 | 4 and 15 |
| 18 | 16 or 17 |
| 19 | (intervent* or program* or strateg* or service* or policy or policies or guideline* or "public health measure*" or treatment* or prevent* or promot* or mitigat* or restrict* or reduc* or support* or implement* or response* or postvention or evaluat*).ti,ab,kw,sh. |
| 20 | 18 and 19 |
| 21 | (education or training or "care coordinat*" or "follow-up care" or aftercare or "media reporting" or hotline* or helpline* or "help line*").ti,ab,kw,sh. |
| 22 | 18 and 21 |
| 23 | (psychopharmac* or medication).ti,ab,kw,sh. |
| 24 | 18 and 23 |
| 25 | *therapy/ or *therapies/ |
| 26 | 18 and 25 |
| 27 | "mental health care".ti,ab,kw,sh. |
| 28 | 18 and 27 |
| 29 | 20 or 22 or 24 or 26 or 28 |
| 30 | limit 29 to english language |
| 31 | limit 30 to humans |
| 32 | remove duplicates from 31 |

**Table S2 Full search strategy Embase (Ovid).**

Databases: Embase Classic+Embase

| **#** | **Searches** |
| --- | --- |
| 1 | (suicid* or self-harm or "self harm" or self-injur* or "self injur*" or parasuicid* or auto-mutilation or automutilat* or "auto mutilat*" or "self mutilat*" or self-immolat* or "self immolat*" or self-poisoning or "self poisoning" or "intentional overdose" or "intentional drug overdose" or self-cutting or "self cutting").mp. [mp=title, abstract, heading word, drug trade name, original title, device manufacturer, drug manufacturer, device trade name, keyword heading word, floating subheading word, candidate term word] |
| 2 | exp suicide/ or suicide.mp. |
| 3 | exp Self-Injurious Behavior/ or self-harm.mp. |
| 4 | 1 or 2 or 3 |
| 5 | (disaster* or catastrophe* or mass-casualty or "mass casualty" or "natural hazard*" or "extreme event*" or "extreme weather event*" or "severe weather" or "heat wave*" or heatwave or drought* or "cold wave*" or cyclone* or hurricane* or tornado* or storm* or cloudburst or flood* or tsunami* or bushfire* or wildfire* or "wild fire" or brushfire or "brush fire" or earthquake* or volcan* or eruption or lahar or "lava flow" or "ash fall" or avalanche* or landslide* or mudslide* or bioevent or bio-event or bio-disaster or "humanitarian emergenc*" or stampede* or terrorism or "terrorist attack*" or "mass violence" or "mass shooting" or "school shooting" or "mass killing*" or explosion* or bombing* or fire or "nuclear accident*" or "nuclear incident*" or "nuclear power plant" or "radiation accident*" or "radiation incident" or CBRNE or CBRN or "oil spill" or "chemical spill" or "structural collapse" or "mine collapse" or "building collapse" or natech or "transport accident*" or "transportation accident*" or "aviation accident*" or "plane crash" or "rail accident*" or derailment or "train crash" or shipwreck or capsize* or "marine incident*" or "financial shock" or "financial crisis" or recession* or "economic downturn*" or "economic depression" or "economic crisis" or austerity).mp. [mp=title, abstract, heading word, drug trade name, original title, device manufacturer, drug manufacturer, device trade name, keyword heading word, floating subheading word, candidate term word] |
| 6 | exp disasters/ |
| 7 | Explosions/ or Fires/ or Structure Collapse/ or Tsunamis/ or Volcanic eruptions/ or Extreme Heat/ or Extreme Weather/ or Biohazard Release/ or Chemical Hazard Release/ or exp Radioactive Hazard Release/ or Accidents, Aviation/ or Accidents, Traffic/ or exp terrorism/ or Bombs/ or Economic Recession/ |
| 8 | 5 or 6 or 7 |
| 9 | ("public health emergenc*" or pandemic* or epidemic* or outbreak*).mp. [mp=title, abstract, heading word, drug trade name, original title, device manufacturer, drug manufacturer, device trade name, keyword heading word, floating subheading word, candidate term word] |
| 10 | Emergencies/ or exp disease outbreaks/ |
| 11 | 9 or 10 |
| 12 | (covid-19 or coronavirus or 2019-ncov or sars-cov-2 or covid* or ncov or hcov or betacoronavirus or Sars-Cov-3 or influenza or H1N1 or H2N2 or H3N2 or H7N9 or "asian flu" or "asian influenza" or "hong kong flu" or "hong kong influenza" or "spanish flu" or "spanish influenza" or ebola or SARS or "severe acute respiratory syndrome" or MERS or Mers-cov or "middle east respiratory syndrome" or "Zika virus" or arbovirus* or dengue or "swine flu" or "swine influenza" or "yellow fever" or "hemorrhagic fever" or "haemorrhagic fever" or encephalitis or plague or smallpox or chikungunya or cholera or "crimean-congo haemorrhagic fever" or "crimean congo haemorrhagic fever" or "crimean-congo hemorrhagic fever" or "crimean congo haemorrhagic fever" or "hendra virus" or "lassa fever" or marburgvirus or marburg virus disease or marburg or meningitis or monkeypox or "nipah virus" or "rift valley fever" or tularemia or tularaemia).mp. [mp=title, abstract, heading word, drug trade name, original title, device manufacturer, drug manufacturer, device trade name, keyword heading word, floating subheading word, candidate term word] |
| 13 | COVID-19/ or Sars-Covid-2/ |
| 14 | 12 or 13 |
| 15 | 11 and 14 |
| 16 | 4 and 8 |
| 17 | 4 and 15 |
| 18 | 16 or 17 |
| 19 | (intervent* or program* or strateg* or service* or policy or policies or guideline* or "public health measure*" or treatment* or prevent* or promot* or mitigat* or restrict* or reduc* or support* or implement* or response* or postvention or evaluat*).ti,ab,kw,sh. |
| 20 | 18 and 19 |
| 21 | (education or training or "care coordinat*" or "follow-up care" or aftercare or "media reporting" or hotline* or helpline* or "help line*").ti,ab,kw,sh. |
| 22 | 18 and 21 |
| 23 | (psychopharmac* or medication).ti,ab,kw,sh. |
| 24 | 18 and 23 |
| 25 | *therapy/ or *therapies/ |
| 26 | 18 and 25 |
| 27 | "mental health care".ti,ab,kw,sh. |
| 28 | 18 and 27 |
| 29 | 20 or 22 or 24 or 26 or 28 |
| 30 | limit 29 to english language |
| 31 | limit 30 to humans |
| 32 | remove duplicates from 31 |

|  |  |  |
| --- | --- | --- |

**Table S3 Full search strategy PsycINFO (Ovid).**

Database: APA PsycInfo

| **#** | **Searches** |
| --- | --- |
| 1 | (suicid* or self-harm or "self harm" or self-injur* or "self injur*" or parasuicid* or auto-mutilation or automutilat* or "auto mutilat*" or "self mutilat*" or self-immolat* or "self immolat*" or self-poisoning or "self poisoning" or "intentional overdose" or "intentional drug overdose" or self-cutting or "self cutting").mp. [mp=title, abstract, heading word, table of contents, key concepts, original title, tests & measures, mesh word] |
| 2 | exp suicide/ or suicide.mp. |
| 3 | exp Self-Injurious Behavior/ or self-harm.mp. |
| 4 | 1 or 2 or 3 |
| 5 | (disaster* or catastrophe* or mass-casualty or "mass casualty" or "natural hazard*" or "extreme event*" or "extreme weather event*" or "severe weather" or "heat wave*" or heatwave or drought* or "cold wave*" or cyclone* or hurricane* or tornado* or storm* or cloudburst or flood* or tsunami* or bushfire* or wildfire* or "wild fire" or brushfire or "brush fire" or earthquake* or volcan* or eruption or lahar or "lava flow" or "ash fall" or avalanche* or landslide* or mudslide* or bioevent or bio-event or bio-disaster or "humanitarian emergenc*" or stampede* or terrorism or "terrorist attack*" or "mass violence" or "mass shooting" or "school shooting" or "mass killing*" or explosion* or bombing* or fire or "nuclear accident*" or "nuclear incident*" or "nuclear power plant" or "radiation accident*" or "radiation incident" or CBRNE or CBRN or "oil spill" or "chemical spill" or "structural collapse" or "mine collapse" or "building collapse" or natech or "transport accident*" or "transportation accident*" or "aviation accident*" or "plane crash" or "rail accident*" or derailment or "train crash" or shipwreck or capsize* or "marine incident*" or "financial shock" or "financial crisis" or recession* or "economic downturn*" or "economic depression" or "economic crisis" or austerity).mp. [mp=title, abstract, heading word, table of contents, key concepts, original title, tests & measures, mesh word] |
| 6 | exp disasters/ |
| 7 | Explosions/ or Fires/ or Structure Collapse/ or Tsunamis/ or Volcanic eruptions/ or Extreme Heat/ or Extreme Weather/ or Biohazard Release/ or Chemical Hazard Release/ or exp Radioactive Hazard Release/ or Accidents, Aviation/ or Accidents, Traffic/ or exp terrorism/ or Bombs/ or Economic Recession/ |
| 8 | 5 or 6 or 7 |
| 9 | ("public health emergenc*" or pandemic* or epidemic* or outbreak*).mp. [mp=title, abstract, heading word, table of contents, key concepts, original title, tests & measures, mesh word] |
| 10 | Emergencies/ or exp disease outbreaks/ |
| 11 | 9 or 10 |
| 12 | (covid-19 or coronavirus or 2019-ncov or sars-cov-2 or covid* or ncov or hcov or betacoronavirus or Sars-Cov-3 or influenza or H1N1 or H2N2 or H3N2 or H7N9 or "asian flu" or "asian influenza" or "hong kong flu" or "hong kong influenza" or "spanish flu" or "spanish influenza" or ebola or SARS or "severe acute respiratory syndrome" or MERS or Mers-cov or "middle east respiratory syndrome" or "Zika virus" or arbovirus* or dengue or "swine flu" or "swine influenza" or "yellow fever" or "hemorrhagic fever" or "haemorrhagic fever" or encephalitis or plague or smallpox or chikungunya or cholera or "crimean-congo haemorrhagic fever" or "crimean congo haemorrhagic fever" or "crimean-congo hemorrhagic fever" or "crimean congo haemorrhagic fever" or "hendra virus" or "lassa fever" or marburgvirus or marburg virus disease or marburg or meningitis or monkeypox or "nipah virus" or "rift valley fever" or tularemia or tularaemia).mp. [mp=title, abstract, heading word, table of contents, key concepts, original title, tests & measures, mesh word] |
| 13 | COVID-19/ or Sars-Covid-2/ |
| 14 | 12 or 13 |
| 15 | 11 and 14 |
| 16 | 4 and 8 |
| 17 | 4 and 15 |
| 18 | 16 or 17 |
| 19 | (intervent* or program* or strateg* or service* or policy or policies or guideline* or "public health measure*" or treatment* or prevent* or promot* or mitigat* or restrict* or reduc* or support* or implement* or response* or postvention or evaluat*).mp. |
| 20 | 18 and 19 |
| 21 | (education or training or "care coordinat*" or "follow-up care" or aftercare or "media reporting" or hotline* or helpline* or "help line*").mp. |
| 22 | 18 and 21 |
| 23 | (psychopharmac* or medication).mp. |
| 24 | 18 and 23 |
| 25 | *therapy/ or *therapies/ |
| 26 | 18 and 25 |
| 27 | "mental health care".mp. |
| 28 | 18 and 27 |
| 29 | 20 or 22 or 24 or 26 or 28 |
| 30 | limit 29 to english language |
| 31 | limit 30 to peer reviewed journal |
| 32 | remove duplicates from 31 |

|  |  |  |
| --- | --- | --- |

**Table S4 Full search strategy Web of Science (Clarivate).**

Database: Web of Science

| **#** | **Searches** |
| --- | --- |
| 1 | TS=(suicid* or self-harm or "self harm" or self-injur* or "self injur*" or parasuicid* or auto-mutilation or automutilat* or "auto mutilat*" or "self mutilat*" or self-immolat* or "self immolat*" or self-poisoning or "self poisoning" or "intentional overdose" or "intentional drug overdose" or self-cutting or "self cutting") |
| 2 | TS=(disaster* or catastrophe* or mass-casualty or "mass casualty" or "natural hazard*" or "extreme event*" or "extreme weather event*" or "severe weather" or "heat wave*" or heatwave or drought* or "cold wave*" or cyclone* or hurricane* or tornado* or storm* or cloudburst or flood* or tsunami* or bushfire* or wildfire* or "wild fire" or brushfire or "brush fire" or earthquake* or volcan* or eruption or lahar or "lava flow" or "ash fall" or avalanche* or landslide* or mudslide* or bioevent or bio-event or bio-disaster or "humanitarian emergenc*" or stampede* or terrorism or "terrorist attack*" or "mass violence" or "mass shooting" or "school shooting" or "mass killing*" or explosion* or bombing* or fire or "nuclear accident*" or "nuclear incident*" or "nuclear power plant" or "radiation accident*" or "radiation incident" or CBRNE or CBRN or "oil spill" or "chemical spill" or "structural collapse" or "mine collapse" or "building collapse" or natech or "transport accident*" or "transportation accident*" or "aviation accident*" or "plane crash" or "rail accident*" or derailment or "train crash" or shipwreck or capsize* or "marine incident*" or "financial shock" or "financial crisis" or recession* or "economic downturn*" or "economic depression" or "economic crisis" or austerity) |
| 3 | TS=("public health emergenc*" or pandemic* or epidemic* or outbreak*) |
| 4 | TS=(covid-19 or coronavirus or 2019-ncov or sars-cov-2 or covid* or ncov or hcov or betacoronavirus or Sars-Cov-3 or influenza or H1N1 or H2N2 or H3N2 or H7N9 or "asian flu" or "asian influenza" or "hong kong flu" or "hong kong influenza" or "spanish flu" or "spanish influenza" or ebola or SARS or "severe acute respiratory syndrome" or MERS or Mers-cov or "middle east respiratory syndrome" or "Zika virus" or arbovirus* or dengue or "swine flu" or "swine influenza" or "yellow fever" or "hemorrhagic fever" or "haemorrhagic fever" or encephalitis or plague or smallpox or chikungunya or cholera or "crimean-congo haemorrhagic fever" or "crimean congo haemorrhagic fever" or "crimean-congo hemorrhagic fever" or "crimean congo haemorrhagic fever" or "hendra virus" or "lassa fever" or marburgvirus or marburg virus disease or marburg or meningitis or monkeypox or "nipah virus" or "rift valley fever" or tularemia or tularaemia) |
| 5 | #3 and #4 |
| 6 | #1 AND #2 |
| 7 | #1 and #5 |
| 8 | #6 or #7 |
| 9 | TS=(intervent* or program* or strateg* or service* or policy or policies or guideline* or "public health measure*" or treatment* or prevent* or promot* or mitigat* or restrict* or reduc* or support* or implement* or response* or postvention or evaluat*) |
| 10 | #8 AND #9 |
| 11 | TS=(education or training or "care coordinat*" or "follow-up care" or aftercare or "media reporting" or hotline* or helpline* or "help line*") |
| 12 | #8 AND #11 |
| 13 | TS=(psychopharmac* or medication) |
| 14 | #8 AND #13 |
| 15 | TS=(*therapy or *therapies) |
| 16 | #8 AND #15 |
| 17 | TS=("mental health care") |
| 18 | **#8 AND #17** |
| 19 | #10 OR #12 OR #14 OR #16 OR #18 |
| 20 | #19 and English (Languages) |

**Table S5 Full search strategy PILOTS/PTSDpubs (ProQuest).**

Database: PTSDpubs

| **#** | **Searches** |
| --- | --- |
| 1 | TI,AB,SU(suicid* or self-harm or "self harm" or self-injur* or "self injur*" or parasuicid* or auto-mutilation or automutilat* or "auto mutilat*" or "self mutilat*" or self-immolat* or "self immolat*" or self-poisoning or "self poisoning" or "intentional overdose" or "intentional drug overdose" or self-cutting or "self cutting") |
| 2 | TI,AB,SU(disaster* or catastrophe* or mass-casualty or "mass casualty" or "natural hazard*" or "extreme event*" or "extreme weather event*" or "severe weather" or "heat wave*" or heatwave or drought* or "cold wave*" or cyclone* or hurricane* or tornado* or storm* or cloudburst or flood* or tsunami* or bushfire* or wildfire* or "wild fire" or brushfire or "brush fire" or earthquake* or volcan* or eruption or lahar or "lava flow" or "ash fall" or avalanche* or landslide* or mudslide* or bioevent or bio-event or bio-disaster or "humanitarian emergenc*" or stampede* or terrorism or "terrorist attack*" or "mass violence" or "mass shooting" or "school shooting" or "mass killing*" or explosion* or bombing* or fire or "nuclear accident*" or "nuclear incident*" or "nuclear power plant" or "radiation accident*" or "radiation incident" or CBRNE or CBRN or "oil spill" or "chemical spill" or "structural collapse" or "mine collapse" or "building collapse" or natech or "transport accident*" or "transportation accident*" or "aviation accident*" or "plane crash" or "rail accident*" or derailment or "train crash" or shipwreck or capsize* or "marine incident*" or "financial shock" or "financial crisis" or recession* or "economic downturn*" or "economic depression" or "economic crisis" or austerity) |
| 3 | TI,AB,SU("public health emergenc*" or pandemic* or epidemic* or outbreak*) |
| 4 | TI,AB,SU(covid-19 or coronavirus or 2019-ncov or sars-cov-2 or covid* or ncov or hcov or betacoronavirus or Sars-Cov-3 or influenza or H1N1 or H2N2 or H3N2 or H7N9 or "asian flu" or "asian influenza" or "hong kong flu" or "hong kong influenza" or "spanish flu" or "spanish influenza" or ebola or SARS or "severe acute respiratory syndrome" or MERS or Mers-cov or "middle east respiratory syndrome" or "Zika virus" or arbovirus* or dengue or "swine flu" or "swine influenza" or "yellow fever" or "hemorrhagic fever" or "haemorrhagic fever" or encephalitis or plague or smallpox or chikungunya or cholera or "crimean-congo haemorrhagic fever" or "crimean congo haemorrhagic fever" or "crimean-congo hemorrhagic fever" or "crimean congo haemorrhagic fever" or "hendra virus" or "lassa fever" or marburgvirus or marburg virus disease or marburg or meningitis or monkeypox or "nipah virus" or "rift valley fever" or tularemia or tularaemia) |
| 5 | 3 and 4 |
| 6 | 1 and 2 |
| 7 | 1 and 5 |
| 8 | 6 or 7 |
| 9 | TI,AB,SU(intervent* or program* or strateg* or service* or policy or policies or guideline* or "public health measure*" or treatment* or prevent* or promot* or mitigat* or restrict* or reduc* or support* or implement* or response* or postvention or evaluat*) |
| 10 | 8 and 9 |
| 11 | TI,AB,SU(education or training or "care coordinat*" or "follow-up care" or aftercare or "media reporting" or hotline* or helpline* or "help line*") |
| 12 | 8 and 11 |
| 13 | TI,AB,SU(psychopharmac* or medication*) |
| 14 | 8 and 13 |
| 15 | TI,AB,SU(therapy or therapies) |
| 16 | 8 and 15 |
| 17 | TI,AB,SU("mental health care") |
| 18 | 8 and 17 |
| 19 | 10 or 12 or 14 or 16 or 18 |
| 20 | limit 19 to Language: English |

**Table S6 Study quality assessment.**

| Quality Criteria | Challinor (2021) | Chapman (2016) | Gujral (2022) | Kim  (2022) | Klim- Conforti (2021) | Lee (2010) | Matsubayashi (2021) | Nakanishi (2020) | Orui (2021) | Vijayakumar (2008) |
| --- | --- | --- | --- | --- | --- | --- | --- | --- | --- | --- |
| **A. Selection bias** | | | | | | | | | |  |
| Representativeness | Somewhat likely | Very likely | Very likely | Very likely | Somewhat likely | Very likely | Very likely | Very likely | Somewhat likely | Very likely |
| Percentage agreed | N/a | N/a | N/a | N/a | < 60% | N/a | N/a | N/a | Can’t tell | 80-100% |
| Rating | Moderate | Strong | Strong | Strong | Weak | Strong | Strong | Strong | Moderate | Strong |
| **B. Study design** | | | | | | | | | |  |
| Study design type | Cohort | Interrupted time series | Cohort | Cohort | RCT | Time series analysis | Cohort | Interrupted time series | Cohort | Cohort analytical |
| Described as randomized? | No | No | No | No | Yes | No | No | No | No | No |
| Method of randomization described? | N/a | N/a | N/a | N/a | Yes | N/a | N/a | N/a | N/a | N/a |
| Method appropriate? | N/a | N/a | N/a | N/a | Yes | N/a | N/a | N/a | N/a | N/a |
| Rating | Moderate | Moderate | Moderate | Moderate | Strong | Moderate | Moderate | Moderate | Moderate | Moderate |
| **C. Confounders** | | | | | | | | | |  |
| Pre-intervention differences? | Can’t tell | Yes | Yes | Can’t tell | No | Yes | Yes | Yes | Yes | Yes |
| Percentage confounders controlled for | Can’t tell | 60-79% (some) | 80-100% (most) | Can’t tell | N/a | < 60% (few or none) | < 60% (few or none) | 60-79% (some) | < 60% (few or none) | < 60% (few or none) |
| Rating | Weak | Moderate | Strong | Weak | Strong | Weak | Weak | Moderate | Weak | Weak |
| **D. Blinding** | | | | | | | | | |  |
| Outcome assessors were blinded? | N/a | N/a | No | N/a | No | N/a | N/a | N/a | N/a | No |
| Participants were blinded? | N/a | N/a | Can’t tell | N/a | No | N/a | N/a | N/a | N/a | Can’t tell |
| Rating | N/a | N/a | Weak | N/a | Weak | N/a | N/a | N/a | N/a | Weak |
| **E. Data collection methods** | | | | | | | | | |  |
| Valid measures? | Can’t tell | Yes | Yes | Yes | Yes | Yes | Yes | Yes | Yes | Yes |
| Reliable measures? | Can’t tell | Can’t tell | Yes | Yes | Yes | Yes | Yes | Yes | Can’t tell | Yes |
| Rating | Weak | Moderate | Strong | Strong | Strong | Strong | Strong | Strong | Moderate | Strong |
| **F. Withdrawals and drop-outs** | | | | | | | | | |  |
| Numbers and reasons reported per group? | N/a | N/a | Can’t tell | N/a | Yes | N/a | N/a | N/a | N/a | Yes |
| Percentage completing study? | 80-100% | N/a | Can’t tell | N/a | 80-100% | N/a | N/a | N/a | N/a | 80-100% |
| Rating | N/a | N/a | Weak | N/a | Strong | N/a | N/a | N/a | N/a | Strong |
| Total A-F: | WEAK | STRONG | WEAK | MODERATE | WEAK | MODERATE | MODERATE | STRONG | MODERATE | WEAK |
| **G. Intervention integrity** | | | | | | | | | |  |
| Percentage participants received intervention? | 80-100% | 80-100% | 80-100% | 80-100% | 80-100% | 80-100% | 80-100% | 80-100% | Can’t tell | 80-100% |
| Intervention consistency measured? | No | Can’t tell | Can’t tell | Can’t tell | Yes | Can’t tell | Can’t tell | No | No | No |
| Confounding unintended intervention? | Can’t tell | Can’t tell | Can’t tell | Can’t tell | Can’t tell | Can’t tell | Can’t tell | Can’t tell | Can’t tell | Can’t tell |
| **H. Analyses** | | | | | | | | | |  |
| Unit of allocation | Individual | Individual | Individual | Community | Organization | Individual | Individual | Individual (family) | Individual | Individual |
| Unit of analysis | Individual | Individual | Individual | Community | Individual | Individual | Individual | Individual | Community | Individual |
| Appropriate statistical methods? | Yes | Yes | Yes | Yes | Yes | Yes | Yes | Yes | Yes | Yes |
| Analysis by intention-to-treat status | N/a | N/a | Can’t tell | N/a | No | N/a | N/a | N/a | N/a | No |

Effective Public Health Practice Project. Quality Assessment Tool For Quantitative Studies. Hamilton: Effective Public Health Practice Project; 1998. https://merst.ca/ephpp/. Accessed 20 August 2023.

**Table S7. Definition of key terms.**

| **Key Term** | **Definition (Source)** |
| --- | --- |
| Disaster | A serious disruption of the functioning of a community or a society at any scale due to hazardous events interacting with conditions of exposure, vulnerability and capacity, leading to one or more of the following: human, material, economic and environmental losses and impacts.  Source: UNDRR <https://www.undrr.org/terminology/disaster> |
| Public Health Emergency of International Concern | An extraordinary event which is determined to constitute a public health risk to other States through the international spread of disease and to potentially require a coordinated international response. This definition implies a situation that is: serious, sudden, unusual or unexpected; carries implications for public health beyond the affected State’s national border; and may require immediate international action.  Source: WHO (2005) International Health Regulations.  <https://www.who.int/news-room/questions-and-answers/item/emergencies-international-health-regulations-and-emergency-committees> |
| Disease Outbreak | A disease outbreak is the occurrence of disease cases in excess of normal expectancy. The number of cases varies according to the disease-causing agent, and the size and type of previous and existing exposure to the agent.  Source: WHO <https://www.who.int/teams/environment-climate-change-and-health/emergencies/disease-outbreaks> |
| Suicide | An action that a person takes to deliberately end their own life, which results in death.  Source: Pirkis et al. (2022). Understanding suicide and self-harm. University of Melbourne.  <https://melbourne.figshare.com/articles/report/Understanding_Suicide_and_Self-harm/24418594> |
| Suicide attempt | An act in which a person harms themselves with the intention of ending their life, and survives.  Source: Pirkis et al. (2022). Understanding suicide and self-harm. University of Melbourne.  <https://melbourne.figshare.com/articles/report/Understanding_Suicide_and_Self-harm/24418594> |
| Self-harm | An act in which a person harms themselves with a motive that may or may not involve the intention of ending their life, and survives.  Source: Pirkis et al. (2022). Understanding suicide and self-harm. University of Melbourne.  <https://melbourne.figshare.com/articles/report/Understanding_Suicide_and_Self-harm/24418594> |
